# Based on quorum sensing: reverse effect of traditional Chinese medicine on bacterial drug resistance mechanism

**DOI:** 10.3389/fcimb.2025.1582003

**Published:** 2025-06-03

**Authors:** Ningning Qiu, Wenlong Liu, Xili Zhang

**Affiliations:** Hunan Key Laboratory of Chinese Medicine Druggability and Preparation, Hunan University of Chinese Medicine, Changsha, China

**Keywords:** bacterial resistance mechanism, quorum sensing, reversal of antibiotic resistance, research progress, Traditional Chinese Medicine

## Abstract

Antimicrobial resistance has emerged as a critical global health challenge requiring urgent multidisciplinary interventions. Pathogenic bacteria utilize six principal resistance mechanisms: (1) Enzymatic degradation of antibiotics via the production of inactivating enzymes; (2) Inactivation of antibiotics by changing the drug targets; (3) Reduction of antibiotics entry by decreasing bacterial permeability; (4) Enhanced antibiotics efflux through overexpression of efflux pumps; (5) Acquisition of antibiotics resistance via genetic mutations; (6) Development of antibiotics resistance through formation of microbial biofilms. Notably, these resistance determinants demonstrate close coordination through quorum sensing, collectively establishing recalcitrant infections that defy conventional therapies. Emerging evidence confirms the therapeutic potential of traditional Chinese medicine in combating antimicrobial resistance. Traditional Chinese medicine can be used as quorum sensing inhibitors to interfere with the quorum sensing of bacteria, thereby achieving antibacterial effects. Moreover, traditional Chinese medicine has the characteristics of rich components, long history, mild action and no drug resistance, which makes it stand out in the research against drug-resistant bacterial infections. This paper systematically describes six mechanisms of bacterial resistance and reviews the antagonistic effects of traditional Chinese medicine against these mechanisms based on quorum sensing. It highlights that the active ingredients, extracts and compound formulations of traditional Chinese medicine have good reversal effects on bacterial antibiotic resistance, which can effectively treat drug-resistant bacterial infections. When combined with antibiotics, traditional Chinese medicine not only reduces antibiotics dosage but also adverse reactions, holding promise for improving and addressing clinical challenges posed by bacterial resistance. This article further discusses the impact of different delivery methods on the anti-bacterial biofilms efficacy of traditional Chinese medicine. It introduces the main delivery methods of traditional Chinese medicine at present and the new delivery methods under research, pointing out the huge development potential in the research of traditional Chinese medicine dosage forms. Additionally, the deficiencies and improvement methods of the current research were pointed out, and prospects for future related research were put forward.

## Introduction

The misuse of antibiotics has intensified the global crisis of bacterial antibiotic resistance, with superbugs posing a significant threat to public health (Liu et al., 2022c). Antimicrobial resistance has become the leading cause of death worldwide, with the largest increase in deaths due to methicillin-resistant *Staphylococcus aureus* (MRSA) infections. For all abbreviations used in this article, please refer to **Supplementary File 1**. Among Gram negative bacteria, the increase in resistance to carbapenem antibiotics is the highest, and antimicrobial resistance is heaviest in resource-limited areas (Christopher et al., 2022; Larsson and Flach, 2022; Sheila et al., 2023), Therefore, deciphering the mechanisms of bacterial resistance and developing strategies to reverse antibiotic resistance are critical imperatives for addressing infections caused by drug-resistant pathogens. As scientific research progresses, the mechanisms underlying bacterial resistance are being progressively elucidated, while strategies to reverse this resistance are undergoing continuous exploration. Bacterial resistance to antibiotics typically arises from antibiotic destruction or modification, target alterations (target substitution, target mutation, target enzymatic alteration, target protection, target overproduction or target bypass), and reduced intracellular antibiotic accumulation due to decreased permeability or increased efflux (Christaki et al., 2020; Denk-Lobnig and Wood, 2023; Smith et al., 2023). Genetic mutation or biofilm formation also represent critical mechanisms of antibiotic resistance (Asenjo et al., 2021; Darby et al., 2023; Grooters et al., 2024). These resistance mechanisms rarely act in isolation; instead, they often involve systemic interactions mediated by quorum sensing among bacteria. As a cellular signal transmission system, quorum sensing generates diverse signal molecules to facilitate intercellular communication, just like weaving a dense network, manipulating the close relationship and interaction between bacterial populations (Liu et al., 2022b; Smith and Schuster, 2022; Su et al., 2023). It is these interwoven relationships that contribute to bacterial resistance. Antibiotics have been hailed as one of the greatest inventions of the 20th century, but now hygiene concerns are forcing people to find new ways to fight them (Behling et al., 2023; Lessa and Sievert, 2023). As a treasure of Chinese cultural heritage, traditional Chinese medicine offers distinct advantages, including diverse botanical resources, high therapeutic value, minimal toxicity, and broad clinical applicability. Traditional Chinese medicine contains rich natural compounds and has great potential for development. In recent years, traditional Chinese medicine has seen extensive applications across industries, including cosmetics, food and healthcare. Popular products such as essential oils, skincare products, functional drinks and milk tea, added with traditional Chinese medicine ingredients, exemplify its versatility. Studies have confirmed that many traditional Chinese medicines have strong antibacterial effect and reverse the bacterial antibiotic resistance. When combined with antibiotics, it can restore the sensitivity of bacteria to antibiotics, offering an effective strategy to mitigate antibiotics resistance. This review summarizes the mechanisms of bacterial drug resistance and discusses the resistance-reversing effects of traditional Chinese medicine through quorum sensing modulation.

## Study on drug resistance mechanism of bacteria

**Figure 1:**
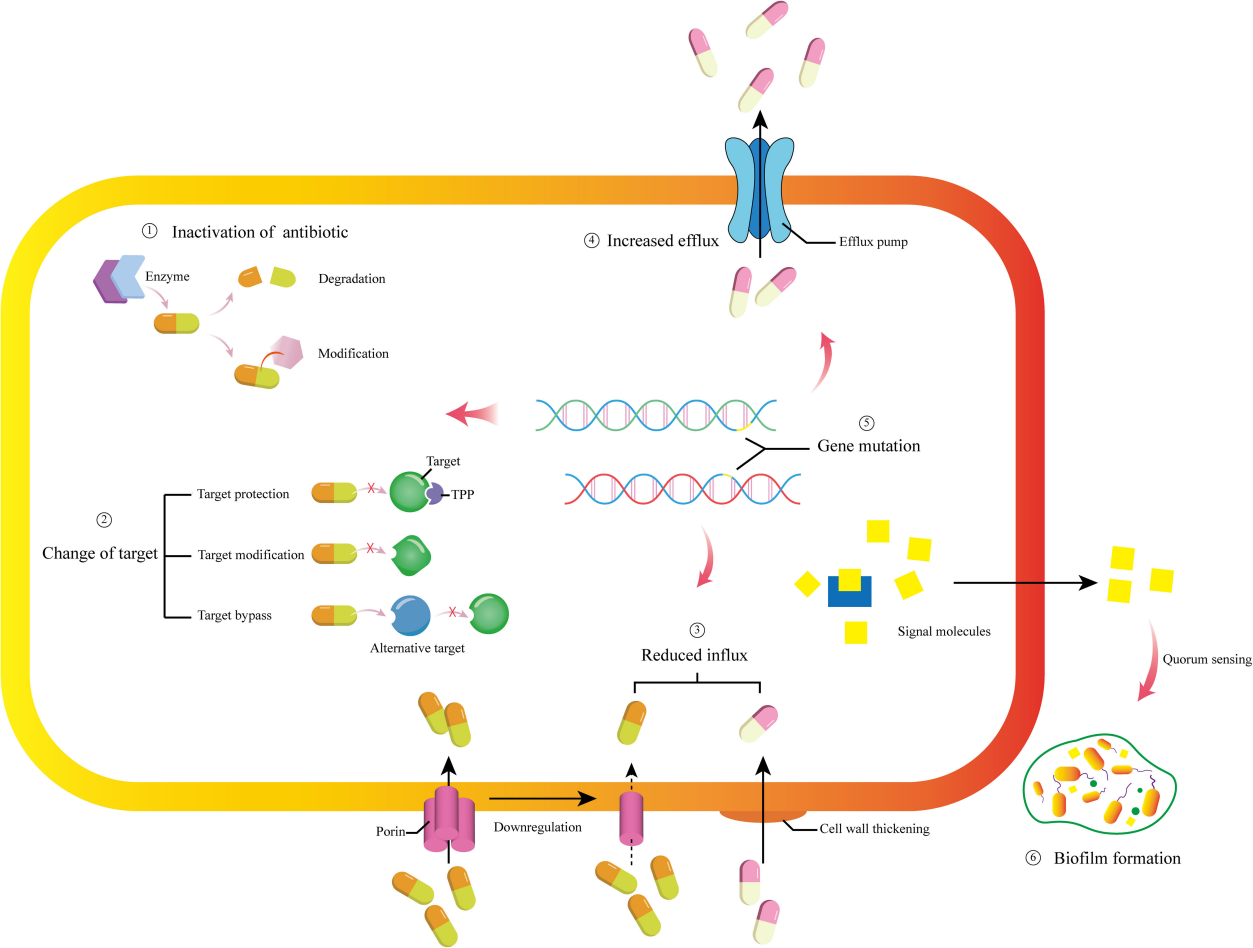
Brief depictions of the molecular mechanism of bacterial resistance. ① Under the enzymatic action of relevant enzymes, antibiotics are degraded and inactivated, or their structures are modified to lose their activity. ② Changing drug targets through three pathways: target protection, target modification, and target bypass. ③ Downregulating the number of porins and thickening the cell wall to reduce the intake of drug molecules. ④ Regulating the efflux pumps to increase the efflux of drug molecules. ⑤ Mutations occur in resistance related gene, and the mutations can regulate other resistance mechanisms. ⑥ Generate signal molecules for quorum sensing, mediating biofilm formation.

At present, typical multidrug-resistant bacteria include *Pseudomonas aeruginosa (P. aeruginosa)*, *Acinetobacter baumannii (A. baumannii)*, MRSA, *Helicobacter pylori (H. pylori)*, etc. The commonalities of these multidrug-resistant bacteria are strong resistance, high virulence, and strong pathogenicity. Their resistance mechanisms are roughly shown in **Figure 1** (Chakraborty et al., 2022; Li et al., 2022; Qin et al., 2022; Darby et al., 2023).

## Production of inactivation enzymes by bacteria

Bacteria develop antibiotic resistance by producing inactivating enzymes or through enzymatic action. Enzymes play a crucial role in the formation of drug resistance via various enzymatic reactions, including hydrolysis, group transfer and oxidation-reduction. For example, β-lactamases hydrolyze β-lactam antibiotics (e.g., penicillin and cephalosporin) by disrupting their four-membered lactam ring, thereby abolishing their biological activity. Similarly, erythromycin esterase inactivates macrolide antibiotics like erythromycin through hydrolysis of their ester bonds (Lima et al., 2020; Mohanty et al., 2021; Michał et al., 2023). Histone acetyltransferase, a member of the N-acetyltransferase family, can transfer acetyl groups to amine groups in a variety of substrates. It can acetylate histones, nuclear high-mobility group proteins, and polyamine isomers, and also exhibits acetylation activity toward kanamycin and streptomycin, two common aminoglycoside antibiotics, resulting in associated resistance (Tomar et al., 2019). Pyruvate kinase, a glycolytic enzyme regulating bacterial metabolism, can be acetylated under the action of non-enzymatic acetyl phosphatase, and its acetylated form is highly expressed in the drug-resistant strains. Studies have demonstrated that acetylation reduces pyruvate kinase activity, enhancing bacterial resistance to antibiotics. Deacetylation can change the conformation of its ATP-binding site, restoring enzyme activity and increasing energy production, thus enhancing the sensitivity of drug-resistant strains to antibiotics (Fang et al., 2022). Tetracycline can be oxidized and inactivated by *TetX* enzymes (Rudra et al., 2018; Cheng et al., 2021), in which purified monooxygenase *MabTetX* can rapidly monooxidize tetracycline and doxycycline, which endows *Mycobacterium abscessus* with high resistance to these two tetracycline. In addition, the monooxygenase *Tet (X4)* endows a variety of bacteria with resistance to tigecycline, and amino acid changes in the *Tet (X4)* structure trigger several alpha helical and beta folds to refold in the second-order structure of the *Tet (X4)* substrate binding domain, thus forming more rings in the structure, making it more flexible and efficient at trapping substrate molecules. The oxidative destruction effect of antibiotics is better, and the resistance is stronger.

## Alteration of drug targets by bacteria

**Figure 2:**
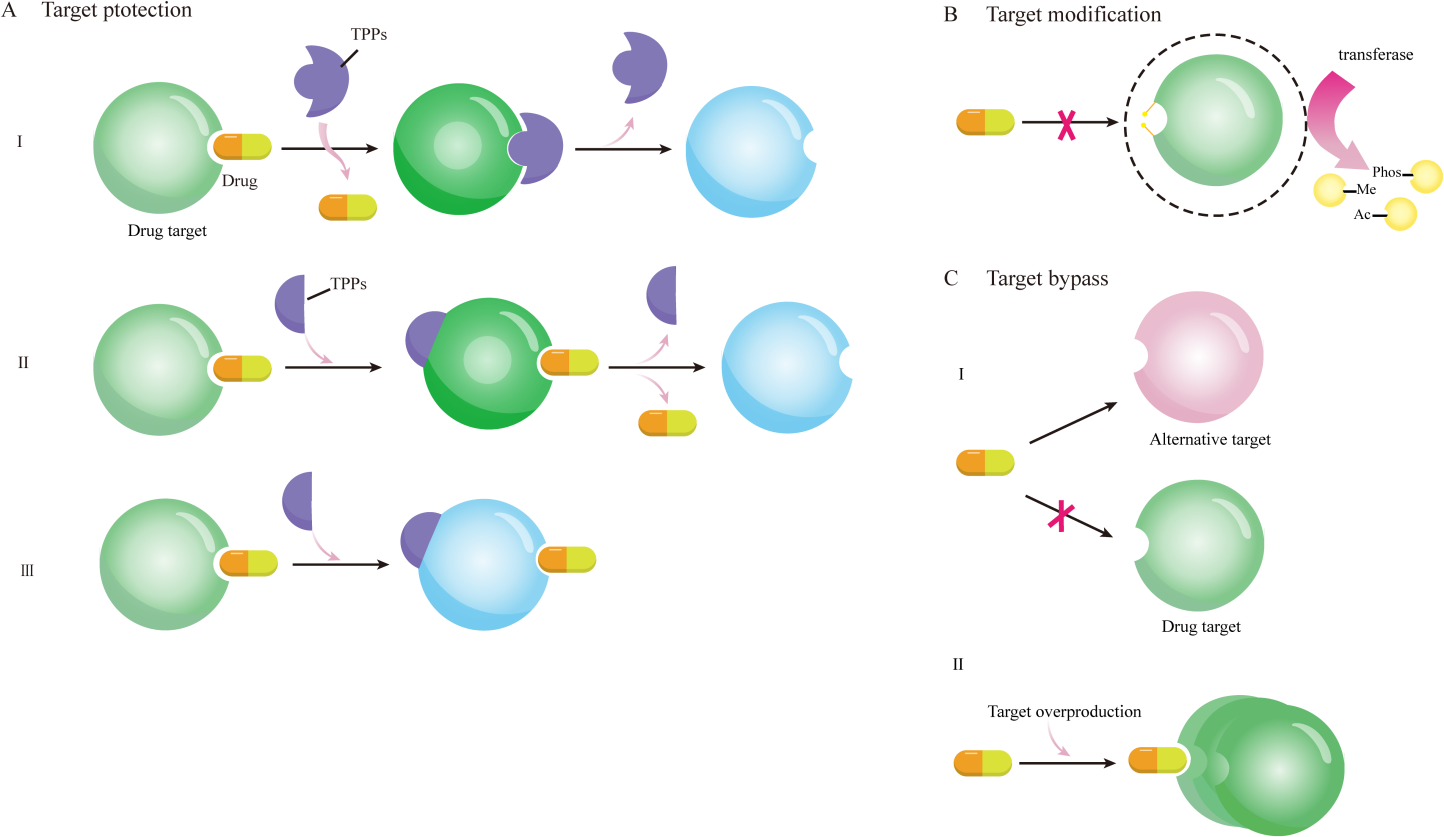
Schematic diagram of drug resistance mechanisms mediated by target protection, target modification, and target bypass. **(A)** shows three ways of target protection: **(I)** TPPs bind to drug targets and remove drugs stereoscopically from the target; **(II)** TPPs bind to drug targets, causing conformational dissociation between drugs and targets; **(III)** TPPs bind to drug targets to cause conformational changes, allowing target proteins to function even in the presence of drugs; **(B)** After the target is modified by various functional groups such as methyl, acetyl and phosphate under the action of transferase, the drug cannot bind to the target to exert its effect; **(C)** alternative targets combine with drugs to resume the function of drug targets, or the overproduction of drug targets make the excess drug targets still function.

Altering drug targets represents a prevalent mechanism of antibiotic resistance (Hunashal et al., 2023; Aleksandrova et al., 2024). For example, polymyxin resistance arises from the morphological changes of lipopolysaccharide (LPS). Mutations in genes encoding the two-component regulatory system—such as those driving *pmrC* overexpression—lead to lipid A modification. When phosphoethanolamine was added to lipid A, the negative charge on LPS and the binding with polymyxin were reduced. The resistance of bacteria to daptomycin was also associated with changes in membrane charge and phospholipid content, the mechanism of drug resistance was due to the redistribution of the cardiophospholipid domain of the main binding site of daptomycin and cocci. Ribosome is an important drug target, and methyltransferase can make bacteria resistant to aminoglycoside antibiotics by modifying the target ribosome with methylylation. After methyltransferase Cfr, which is expressed in thermophilic bacteria, methylates adenine residues 2503 in the ribosome center, nucleotide A2062 is also allosteric rearrangement, which likely prevents catalytic peptidyl transferase-targeting antibiotics from binding to the ribosome. This mechanism underscores the drug resistance caused by methyltransferase Cfr. Tetracycline drugs inhibit bacterial protein synthesis by binding to the 30S subunit of ribosomes, and *Staphylococcus aureus (S. aureus)* circumvents tetracycline activity through targets protection mechanisms, primarily involving Tet(M) and Tet(S) proteins that dissociate tetracycline molecules from the 30S subunit of the ribosome (Mlynarczyk-Bonikowska et al., 2022). Target protection plays a key role in clinical antibiotic resistance (Wilson et al., 2020), and Target protective proteins (TPPs) can mediate antibiotic resistance in three ways: remove drugs sterically from the targets (class I), separate the drugs from the targets allosterically by inducing a structural change within the target (class II), or restore the function of the targets in the presence of binding antibiotics by inducing a conformational change within the target (Class III),as shown in **Figure 2** (Darby et al., 2023; Xiao et al., 2023).

## Reduction of cellular permeability in bacteria

Gram negative bacteria can synthesize various specific Outer membrane porins (Omps). Hydrophilic antibiotics (such as lactams, fluoroquinolones, and tetracyclines) are difficult to pass through the lipid bilayer, rely on Omps for cellular entry to exert antibacterial effects. Therefore, loss or impairment of Omps, or reduced bacterial membrane permeability via changes in the properties and quantity of porins can lead to bacterial resistance (Zhang and Cheng, 2022; Gauba and Rahman, 2023). For example, *P. aeruginosa* expresses specific porins, including OprD (an alkaline amino acid-specific porin with a carbapenem-binding site critical for antibiotic uptake). The absence of OprD in *P. aeruginosa* is a key driver of its resistance phenotype (Pang et al., 2019). In *Klebsiella pneumoniae* (*K. pneumoniae*), point mutations or insertions in the coding sequences or promoter regions of the major general diffusion porins OmpK35 and OmpK36 lead to their loss, conferring resistance to beta lactams and fluoroquinolones (Khalid et al., 2020). Gram positive bacteria can alter gene expression levels by coordinating the action of tyrosine kinase WalK and response regulatory factor WalR. For example, overexpression of *glmU* and *murG* involved in cell wall metabolism regulation enhances peptidoglycan synthesis in *Staphylococcus* species, thickening the cell wall and impeding antibiotic penetration, thereby increasing the resistance (Baran et al., 2023).

## Enhanced drug efflux in bacteria

A well-known mechanism of drug resistance is the overexpression of AcrAB-TolC pumps caused by mutations in the regulatory gene coding region. AcrAB-TolC efflux pumps belong to resistant nodule differentiation (RND) family efflux pumps. They can mediates antibiotic efflux across the bacterial inner membrane, periplasmic space, and outer membrane into the extracellular environment (Plé et al., 2022; Vieira Da Cruz et al., 2024). Previous studies have shown (Hornsey et al., 2010) that efflux pumps such as mexXY, AcrAB, and OqxAB are often associated with bacterial multidrug resistance. The decreased sensitivity of *Enterococcus cloacae* to tigecycline is the result of *ramA* mediated overexpression of AcrAB efflux pumps; The reduced sensitivity of *K. pneumoniae* to tigecycline is also related to the structural overexpression of mexXY and AcrAB. In a tracking study of clinical strains of *K. pneumoniae* (Ye et al., 2017), it was found that the inhibition of the ribosome binding site sequence led to the abolition of *RamR* translation and the disruption of *RamR*’s inhibitory effect on *RamA*, resulting in an increase in the levels of r*amA* and AcrAB, which contributes to the development of bacterial resistance. When the concentration of antibiotics in *Escherichia coli (E. coli)* increases, the outer membrane protein TolC can transiently bind to AcrAB, MacAB, and EmrAB proteins, forming a tripartite efflux pump component that spans the entire cytoplasmic space and can expel harmful substances from the cytoplasm to the extracellular region (Kantarcioglu et al., 2024). Research has also revealed that RNA can regulate drug efflux to develop drug resistance. Under reatment with the antituberculosis drug rifampicin, the number of small RNA MTS1438 in *Mycobacterium tuberculosis (M. tuberculosis)* is significantly upregulated. MTS1438 upregulates the efflux protein CydC, causing rifampicin to flow out of cells, resulting in higher abundance of MTS1438 in cells, less drug accumulation, and stronger drug resistance (Singh and Dutta, 2024).

## Gene mutations in bacteria

*M. tuberculosis* can acquire drug resistance through mutations in the *gyrA* and *gyrB* genes, which affect DNA replication. Among them, *gyrA* is a hotspot region for mutations, termed the quinolone resistance determining region (QRDR). Mutations in this region often lead to resistance to quinolone drugs. The *rpoB* gene mutation can prevent rifampicin from binding to the RNA polymerase beta subunit, thereby blocking drug-mediated inhibition of bacterial transcription and conferring drug resistance. Aminoglycoside drugs exert bactericidal effects mainly by inhibiting bacterial protein synthesis by combining with ribosome. The codon 43 mutation in rpsL (encoding ribosomal protein S12), which converts lysine to arginine, disrupts the structure of 16S rRNA, interrupt the interaction between 16S rRNA and streptomycin, and make *M. tuberculosis* resistant to streptomycin. Similarly, *rpsL* substitution mutations are also responsible for high-level streptomycin resistance in *Yersinia pestis.* In addition, mutations in the rrs gene (encoding 16S rRNA) have been implicated in streptomycin resistance in M. tuberculosis, while rrs mutations can also confer kanamycin resistance. The *tlyA* gene mutation render *M. tuberculosis* resistant to polypeptide antibiotics that inhibit the protein synthesis of them, thereby complicating tuberculosis treatment (Dai et al., 2021; Shafipour et al., 2022; Hou et al., 2024). What’s more, mutations in genes related to central carbon and energy metabolism can reduce bacterial basal respiration, thereby preventing antibiotics-mediated induction of tricarboxylic acid cycle activity and evading metabolic toxicity (Lopatkin et al., 2021). In addition, reduced tricarboxylic acid cycle activity promotes lipid anabolic metabolism, leading to increased lipid anabolic metabolism, and thickened cell wall, thus reducing drug sensitivity (Goossens et al., 2020).

## Biofilm formation by bacteria

Biofilm is a protective membrane composed of Extracellular polymeric substances (EPS), such as polysaccharides and proteins, constantly secreted by bacterial clusters. Its growth is regulated by quorum sensing, and bacteria are enveloped in it to withstand environmental pressure and drug attacks. Biofilm (Karygianni et al., 2020; Buzzo et al., 2021; Du Toit, 2024; Li et al., 2024) form a protective barrier that antibiotics cannot penetrate. The EPS produced by bacteria can create a unique microenvironment for cells within the biofilm by inducing potential oxygen gradients or signaling nutrient limitations. Cellulose and curli pili act synergistically in the biofilm as scaffolds, mediating surface adhesion and tightly stacking cells within hydrophobic networks, which endows cells with resistance to the antibiotic by restricting antibiotics penetration. For example, extracellular DNA in the matrix imposes cationic restrictions on cells within the biofilm, increasing antibiotic resistance in the biofilm. Therefore, the drug resistance of biofilms can be partially attributed to both the physical barrier formed by matrix polymers and the microenvironment stress induced by high-density cell proliferation within the matrix (MacKenzie et al., 2017; Aleksandrowicz et al., 2023).

Edeer et al. (Montoya-Hinojosa et al., 2024) reported that planktonic bacteria exhibit moderate resistance to trimethoprim sulfamethoxazole but low resistance to chloramphenicol, minocycline, levofloxacin cefotaxime, and meropenem. In contrast, bacterial biofilms formed by these bacteria display high resistance to the same antibiotics, requiring higher concentrations of antibiotics for eradication. When the populations of planktonic *E. Coli* and biofilm *E. Coli* were exposed to the antibiotic amikacin for several weeks, biofilm-forming E. coli rapidly acquired mutations in genes encoding transporters and elongation factors. This elevated mutation rate drove the enhancement of drug resistance, a phenomenon not observed in planktonic cells. These findings highlight that the biofilm microenvironment facilitates rapid antibiotic resistance evolution (Usui et al., 2023). (Rahmoun et al., 2021) compared the biofilm-forming capabilities across *Clostridium difficile* strains and identified a positive correlation between reduction of antibiotic sensitivity and enhancement of biofilm production capacity. Specifically, stronger biofilm-forming capacity was associated with greater bacterial resistance.

## Quorum sensing in bacteria

**Figure 3:**
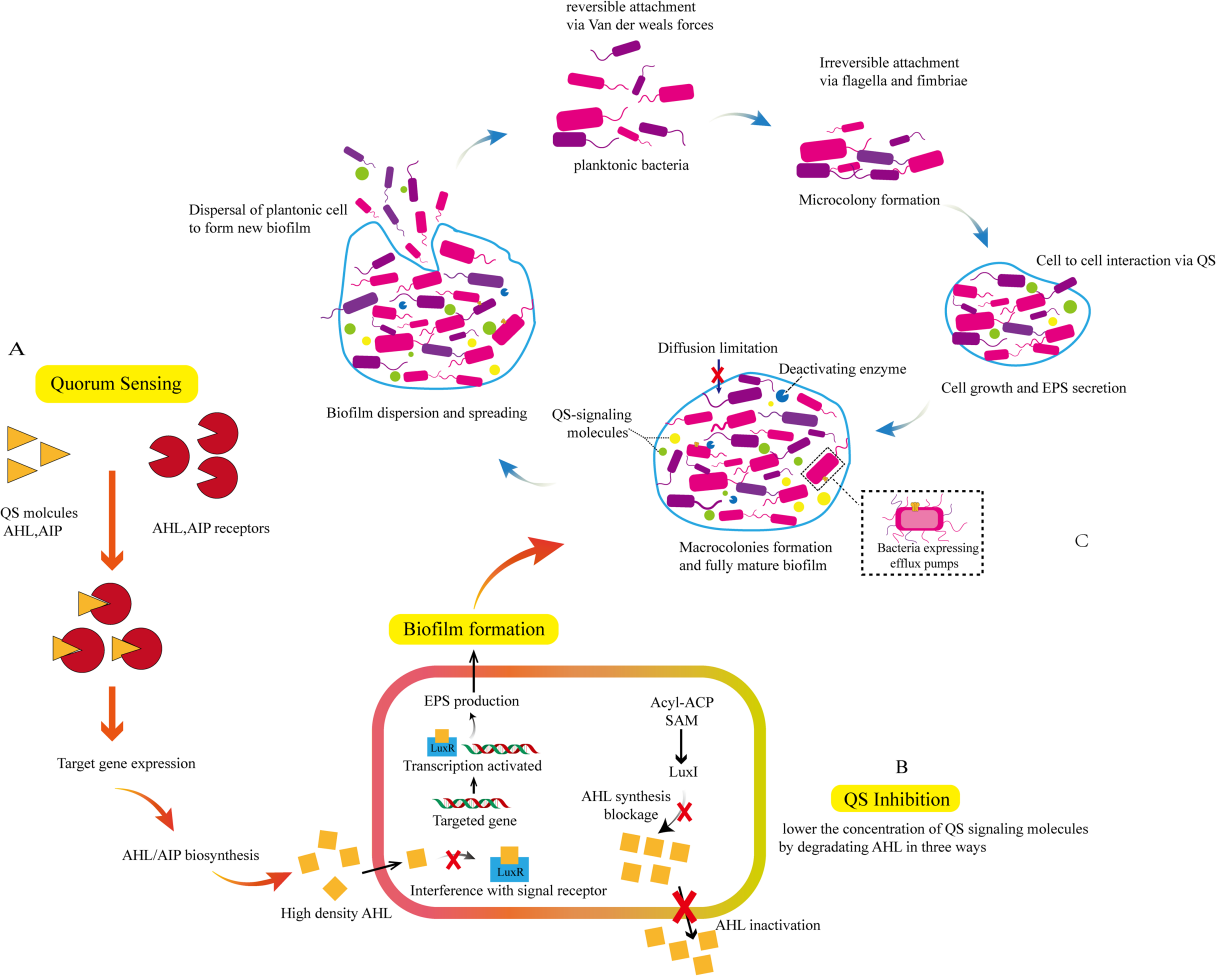
The mechanism by which QS signaling molecules mediate the generation of bacterial biofilms. Signal molecules such as Acyl - homoserine lactones (AHL) and Autoinducing Peptides (AIP) bind to corresponding receptors to induce targeted gene expression and promote the generation of signal molecules, as shown in **(A)**; Signal molecules are transmitted between cells to assist bacteria in QS. Reducing the concentration of signal molecule AHL can inhibit QS. The degradation of AHL includes: (1) interfering with AHL signal receptors (2) blocking AHL biosynthesis(3) inactivating AHL; as shown in **(B)** Signal molecules induce target gene expression, transcription, and generation of EPS, promoting the formation of biofilms. The formation of biofilms includes: (1) reversible binding of planktonic bacteria via van der Weals forces (2) irreversible binding of bacteria via flagella and fimbriae and forming microcolonies (3) transmission of signal molecules between bacterial microcolonies, and interaction between cells via QS. During this stage, bacteria continue to grow and secrete EPS, and the biofilm begins to take shape (4) formation of macrocolonies and fully mature biofilms (5) dispersal of mature biofilms and the spread of planktonic bacteria to generate new biofilms, as shown in **(B, C)**.

Quorum sensing is a chemical communication process used by bacteria to coordinate group behavior, involving the production, release, and population-wide detection of signaling molecules termed autoinducer. It will be replaced by QS in the following text. QS enables bacterial communities to synchronously modulate behaviors in response to fluctuations in population density and species composition of neighboring communities (Eickhoff and Bassler, 2018; Mukherjee and Bassler, 2019). Its coordinated behaviors in cellular populations includes bioluminescence, production of virulence factors, secondary metabolism, biofilm formation, etc. The mechanism of bacterial QS signaling molecules mediating biofilm formation can be referred to **Figure 3** (Sahli et al., 2022; Sahreen et al., 2022). In the flow experiment of *S. aureus*, the pathogen can leverages flow-induced silencing of peripheral cell QS to form a robust biofilm, while allowing cells at the bottom of the biofilm to escape and establish new colonies elsewhere, thereby safeguarding the bacterial community (Hofer, 2016). The genes encoding extracellular product generation are upregulated under QS regulation, and *P. aeruginosa* can produce a series of tissue-damaging extracellular products, including proteases that facilitate its dissemination within host tissues. Notably, many of these extracellular products are generated by QS regulation (Azimi et al., 2020). Collectively, QS systems contribute to the expression of bacterial virulence factors and facilitate the development of bacterial resistance. Therefore, targeting QS to suppress bacterial resistance represents a promising strategy for treating bacterial infections (Fan et al., 2022; Su and Ding, 2023; Vashistha et al., 2023; Zhang et al., 2024b). For example, the signaling molecule autoinducers-2 (AI2) in QS plays an important role in interspecies bacterial communication and resistance development, coordinating bacterial biofilm formation and virulence factors production (Zhang et al., 2020; Ramić et al., 2022). The AI-2 inhibitory compound Str7410 can inhibit bacterial QS activity, not only significantly impeding mixed-species biofilm formation but also enhancing the susceptibility of bacterial consortia to antibiotics when used in combination. This synergistic effect is attributed to Str7410’s ability to reduce the production of virulence factors (e.g., pyocyanin, elastase) and downregulate the expression of QS-related genes (Jiang et al., 2022). In addition, molecularly imprinted polymers can be developed to capture prototype QS autoinducer, thereby interrupting QS and inhibiting biofilm formation of *P. aeruginosa*. Molecularly imprinted polymers hold promise as biofilm-intervening agents for clinical environments and the surfaces of food-processing equipment (Ma et al., 2018). Both signal molecule inhibitors and biofilm-intervening strategies offer innovative ideas for treating multidrug-resistant bacterial infections: Inhibiting the QS system represents a critical breakthrough in addressing bacterial resistance. Research has demonstrated (Zhang et al., 2023; Wang et al., 2024) that the combination of quorum sensing inhibitors (QSIs) and antibiotics can improve bactericidal efficiency and avoid antibiotic resistance caused by excessive use of antibiotics.

## Traditional Chinese medicine inhibits quorum sensing to combat bacterial resistance mechanisms

QS plays a crucial role in bacterial drug resistance. With the escalating crisis of antimicrobial resistance, the therapeutic efficacy of conventional antibiotics alone has gradually diminished. Most traditional Chinese medicines can function as QSI targeting the QS system, reversing bacterial drug resistance, enhancing the efficacy of antibiotics, and helping to alleviate the clinical drug resistance predicament. Traditional Chinese medicine as QSI has the following advantages: 1. Chinese medicinal materials are rich in components and contain various active ingredients. Their extracts can act on multiple links of the QS system, interfere with QS through multiple pathways to combat bacterial resistance, and also provide resources for the development of new QSI. Traditional Chinese medicine compound prescriptions have a long history and rich experience in medication, and can provide guidance for the screening and development of potential QSI. 2. Compared with chemical drugs, most traditional Chinese medicines have milder effects on the human body, fewer toxic and side effects, higher safety. They are more accessible than chemical drugs, and less likely to develop drug resistance. Their development as QSI has obvious advantages. At present, screening QSI from traditional Chinese medicine resources has become a new strategy for developing natural antibacterial drugs (Yang et al., 2021; Zhao et al., 2021; Farha et al., 2023; Fu et al., 2024).

## Active ingredients of traditional Chinese medicine

The licorice flavonoids in traditional Chinese medicine *Glycyrrhiza uralensis* can effectively inhibit the QS system and reduce the virulence of *A. baumannii* by downregulating the expression of the autoinducer synthase gene *abaI*. Among them, Glabridin exhibits dose-dependent inhibition of the motility and biofilm formation ability of multidrug-resistant *A. baumannii.* Its mechanism involves downregulating the autoinducer synthase genes *abaI* and *abaR*, inhibiting the generation of AHL in the QS system, and consequently disrupting the formation of *abaR*-AHL complexes (Lin et al., 2023). Chlorogenic acid demonstrates analogous effects on multidrug-resistant *A. baumannii*. Under the treatment of chlorogenic acid, the expression of QS-related genes in bacteria was also downregulated (Xu et al., 2022). Chlorogenic acid eliminates multidrug-resistant *P. aeruginosa* by inhibiting bacterial movement, reducing pyocyanin production, suppressing elastase activity, and inhibiting biofilm formation. Nine monomers of traditional Chinese medicine, including caffeic acid, cinnamic acid, and myricetin, can also combat *A. baumannii* infections by downregulating QS genes, inhibiting bacterial QS, and inhibiting the expression of bacterial virulence factors (Zeng et al., 2023). Among them, chlorogenic acid is widely present in traditional Chinese medicinal materials of the genus Lonicera in the family Caprifoliaceae and the genus Artemisia in the family Asteraceae. Traditional Chinese medicinal materials with relatively high chlorogenic acid content include *Eucommia ulmoides*, *Lonicera japonica*, *Chrysanthemum morifolium*, and *Houttuynia cordata*, all of which are commonly used. The QS system can regulate the formation of bacterial biofilm, and the ability of bacterial biofilm formation is positively correlated with bacterial resistance. Studies have shown that berberine and matrine can inhibit biofilm formation and weaken bacterial resistance by suppressing the QS system of *E. coli*, and berberine is more effective than matrine (Sun et al., 2019). Berberine mainly comes from the traditional Chinese medicines *Coptis chinensis* and *Phellodendron amurense*, while matrine mainly exists in the traditional Chinese medicine *Sophora flavescens*. Antimicrobial photodynamic therapy (APDT) can effectively eradicates biofilms,and Curcumin (Abdulrahman et al., 2020; Xia et al., 2023) mediated APDT can inhibit the expression of QS pathway related genes, and the inhibition intensity varies under different light conditions. Upon increasing the light dose to 10J/cm^2^, QS gene expression levels decline sharply, resulting in a significant decrease in the thickness of *P. aeruginosa* biofilm and the production of EPS. Therefore, curcumin mediated APDT may be a potential therapeutic method for controlling biofilm mediated infections. Curcumin is the primary active component of the traditional Chinese medicine *Curcuma longa*. Besides, the traditional Chinese medicines Curcuma wenyujin and Curcuma zedoaria are also important sources of curcumin. The non cyclic monophenolic compound geraniol is widely present in various Chinese medicinal materials such as *Citrus reticulata*, *Rosa rugosa*, and *Cymbopogon citratus*. Studies have found that geraniol (Li et al., 2023a) can inhibit the expression of key genes in the QS system of *P. aeruginosa* PAO1, including the signal synthase-coding genes *lasI*, *rhlI*, and *pqsABCDEH*, the corresponding signal receptor coding genes *lasR*, *rhlR*,*pqsR*, as well as virulence genes including *rhlABC*, *lasAB*, *lecAB*, *phzABMS*, and *pelABG*, which weaken virulence factors such as rhamnolipids, proteases, and biofilms, helping to reduce the resistance of *P. aeruginosa* PAO1. The natural furan coumarin compound psoralen (Wen et al., 2024) can inhibit most QS activated genes and virulence factors, with the strongest inhibition observed in the Rhl system. When combined with levofloxacin, kanamycin, etc., psoralen exhibits synergistic antibacterial activity and enhances the sensitivity of *P. aeruginosa* to antibiotics. Psoralen is mainly derived from *Psoralea corylifolia*, a leguminous plant.

**Figure 4:**
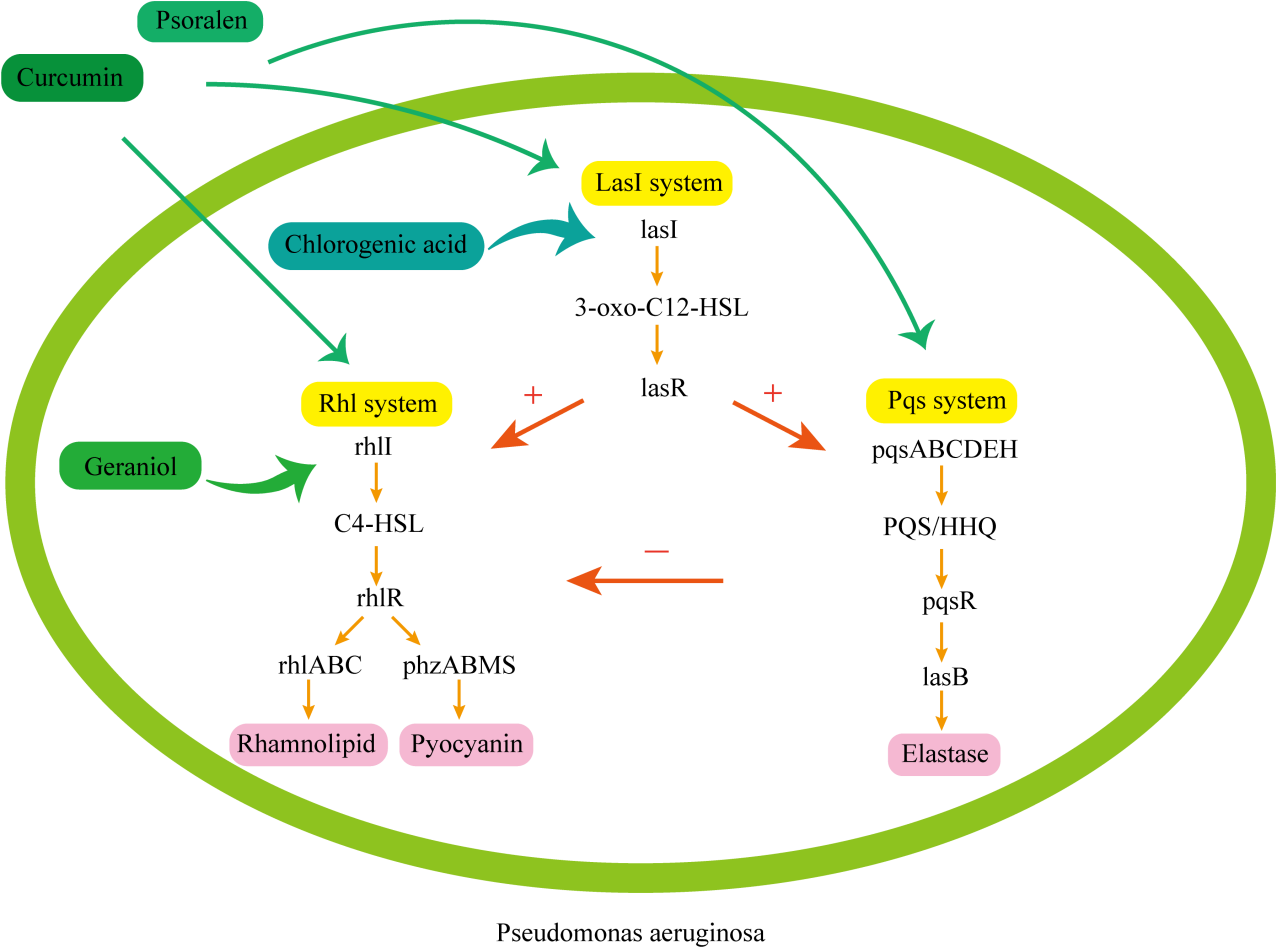
Common QS pathways in *P. aeruginosa.* 1. Las system: The lasI gene encodes autoinducer synthase generation, generating the signal molecule 3-oxo-C12-HSL and lasR binds to the signal molecule, activating the target gene. The Las system can activate the Rhl system and the Pqs system. 2. Rhl system: The rhlI gene encodes related enzymes to produce the signal molecule C4-HSL and rhlR binds to the signal molecule, activating the target gene which regulates the generation of virulence factors such as rhamnolipid and pyocyanin. 3. Pqs system: The pqsABCDEH gene encodes the synthesis of the signal molecules PQS and HHQ. pqsR binds to the signal molecules, activating the virulence gene, which regulates the generation of the virulence factor elastase. Chlorogenic acid mainly acts on the Las system. It inhibits LasI synthase to reduce the generation of the signal molecule 3-oxo-C12-HSL, and affects the generation of subsequent virulence factors. Curcumin and psoralen can act widely on three systems. Curcumin mainly affects the expression of virulence factors by inhibiting the synthase of signal molecules, reducing the production of signal molecules and the expression of receptor genes, while psoralen can simultaneously inhibit the expression and transcription of receptor genes lasR, rhlR, and pqsR in the three systems. Geranol mainly acts on the Rhl system. It inhibits RhlI synthase, reduces the synthesis of the signal molecule C4-HSL, and blocks the generation of virulence factors.

The common QS pathways involved in some of the mentioned active ingredients are shown in **Figure 4** (Liu et al., 2022a).

## Traditional Chinese herbal extracts

Herbal extracts made from *Artemisia argyi*, *Dictamni cortex*, and the root of *Solanum melongena* can target and inhibit the Pqs system in the QS system of *P. aeruginosa*. This herbal extract can act as a competitive agent to inhibit the binding of MvfR to the corresponding *pqsA* promoter region, thereby suppressing bacterial virulence and quenching QS function in *P. aeruginosa*. Its effect on the Las and Rhl systems is relatively small (Wei et al., 2020). Essential oils extracted from traditional Chinese medicinal materials of the family citrus (*e.g., Citrus limon, Citrus paradisi*) are rich in terpenes such as limonene, which can reduce the synthesis of QS signals, proteases, and pyocyanins in *P. aeruginosa*, inhibiting bacterial movement and biofilm formation (María Constanza et al., 2016; Luciardi et al., 2021). The antibacterial mechanism of *Cinnamomum cassia* leaf ethanol extract (Alva et al., 2021) against *P. aeruginosa* is similar. Multiple extract components, including aniline, Cyclohexyl-15-crown-5, 2-Acrylic acid, etc., exhibit anti-QS activity, which can serve as potential QSIs for the treatment of drug-resistant bacterial infections. Extracts of *Cassia fistula* (Peerzada et al., 2022) and *Artemisia annua* leaves (Khan et al., 2024) are also potential QSIs, which can significantly inhibit the production of virulence factors (e.g., anthocyanins, pyocyanins, proteases, chitinase, rhamnolipids) and biofilm formation in *P. aeruginosa* PAO1, thereby combating bacterial resistance. *Scutellaria baicalensis* contains abundant flavonoids such as baicalin and baicalein (Wang et al., 2021b; Zhang et al., 2021), which can interfere with the synthesis of Pqs molecules by regulating *pqsA* and *pqsR*, downregulate the expression of QS-related genes, reduce many important virulence factors in *P. aeruginosa*, including the type III secretion system, diminish cytotoxicity, and accelerate bacterial clearance. Studies have found that *Ginkgo biloba* peel extracts (Wang et al., 2021a, Wang et al., 2022) can inhibit the biofilms formation in *S. aureus* and *Staphylococcus haemolyticus* by regulating relevant genes, and it demonstrates broad-spectrum antibacterial activity against both Gram positive and Gram negative bacteria. *Panax ginseng* (Wang et al., 2020), rich in active substances such as ginsenosides, polysaccharides, proteins, and panaxatriols, exerts antibacterial effects against multidrug-resistant bacteria (e.g., *H. pylori*, *S. aureus*, *E. coli*) by inhibiting microbial motility and QS ability, affecting biofilm formation and destroying mature biofilm, thereby reducing the infection of microorganisms. Extracts of *Coptis chinensis* and *Schisandra chinensis* have inhibitory effects on biofilm formation, motility, and expression of virulence factors in *Vibrio alginolyticus* (Yi et al., 2023; Zhu et al., 2024), with mechanisms potentially involving downregulation of QS-related genes.

## Traditional Chinese medicine compound

The traditional Chinese medicine compound Tanreqing (TRQ) can inhibit the expression of upstream regulatory factors in the QS system of *P. aeruginosa*, including the two-component systems GacS/GacA and Ppra/PprB, as well as the production of virulence factors such as pyocyanin, rhamnolipids, elastase, and alkaline protease (Yang et al., 2020). Notably, TRQ demonstrates potent reversal effects on the resistance of multidrug-resistant *P. aeruginosa*. *S. aureus* utilizes diffusible autoinducers for intercellular communication and generates biofilms triggered by AI-2 (Sedlmayer et al., 2021), contributing to its robust multidrug resistance. TRQ can resist *S. aureus* infections by modulating QS-related genes and downregulating the expression of virulence factors such as hemolysin and autolysin (Yang et al., 2022b). When combined with vancomycin or linezolid, TRQ can significantly enhance the anti biofilm efficacy against MRSA, increase bacterial sensitivity to antibiotics, and reduce required antibiotic dosage (Yang et al., 2018). In *H. pylori* (Yang et al., 2022a), the QS signaling molecule AI-2 induces expression of urease structural proteins UreA and UreB, enabling the pathogen to produce large amounts of urease for acidic environment adaptation and gastric survival. Orphan response regulator HP1021 can directly bind to UreAB to inhibit urease expression, while HP1021 can be downregulated by AI-2 to restore urease expression. As bacterial density increases, AI-2 mediated QS enhances acid tolerance and facilitates bacterial dissemination following gastric acid exposure. Therefore, inhibiting the QS system to reduce biofilm formation and urease production (Gopalakrishnan et al., 2020) may effectively combat multidrug-resistant *H. pylori* infections. For example, Chinese patent medicine Jinghua Weikang capsule can reverse the resistance of *H. pylori* to metronidazole by reducing the adsorption of *H. pylori* to gastric mucosal epithelial cells and inhibiting the formation of biofilm (Jia et al., 2022). Banxia Xiexin Tang contains multiple ingredients inhibiting *H. pylori*, such as berberine, emodin, and luteolin, which can activate immune function, reduce virulence factors like urease of *H. pylori*, and enhance bactericidal effects. Its mechanism may involve inhibiting AI-2 activity in the QS system, with superior therapeutic efficacy and safety compared to triple therapy for *H. pylori* infections (Mei et al., 2019; Li et al., 2023b). When combined with triple therapy, it can reduce antibiotic dosage, enhance efficacy, and mitigate resistance development. Xuebijing can inhibit biofilm formation of *P. aeruginosa*, downregulate the expression of QS-related genes, involving Las, Rhl, Pqs systems, and the virulence factors, to combat pathogenic bacterial infections. The combination of Qiguiyin compound (Chen et al., 2022) and antibiotics can treat multidrug-resistant *P. aeruginosa* infections, enhancing antibiotic efficacy. Serum proteomics revealed 76 differentially expressed proteins in rats before/after Qiguiyin compound treatment. Proteins including PhzA, PhzB, PhzM, MetQ1 can promote QS and biofilm formation in *P. aeruginosa* and were downregulated after treatment. Qiguiyin compound affects the drug resistance of *P. aeruginosa* by modulating protein expression, with these proteins individually or synergistically participate in regulating QS, secretion system, and biofilm formation.

## Effect of different delivery methods of traditional Chinese medicines on their anti-biofilm ability

QS is intricately linked to the formation of biofilms, and the eradication of biofilms remains a thorny issue in clinical treatment. Different delivery methods of drugs have different eradication effects on bacterial biofilms. Currently, the delivery method of most traditional Chinese medicines rely on oral delivery, with common dosage forms including pills, granules, capsules, decoctions, etc (Wu et al., 2023). However, oral delivery often suffers from low bioavailability and limited efficacy. Therefore, the development of traditional Chinese medicine dosage forms has broad prospects. The traditional Chinese medicine compound TRQ comes in various dosage forms such as capsules, granules, injections, and gels. Currently, the most commonly used method in clinical practice is intravenous injection of TRQ. Compared with oral delivery and local delivery, injection delivery enables the active drug to enter the bloodstream more rapidly. It can exert the efficacy of TRQ in inhibiting the biofilm of *K. pneumoniae* more fully (Zhang et al., 2024a). Nanotechnology has emerged as a promising delivery method. The advantages of nanoparticles, such as small volume and large surface area, facilitate their penetration into the biofilm matrix. Surface-modified ligands enable receptor-targeted binding, facilitating localized drug release and superior anti-biofilm activity (To et al., 2023). Nanodelivery systems not only improve drug stability but also overcome physiological barriers through altering surface modification, expanding therapeutic applications, which make them excellent delivery methods for enhancing drug efficacy. The shiborin extracted from the traditional Chinese medicine *Radix lithospermi* has poor water solubility (Sun et al., 2022) and low bioavailability for oral delivery. Making it into shiborin nanoemulsion and shiborin-Fe nanoparticles can address the problems of poor water solubility and light stability, enhancing shikonin’s eradication efficacy against biofilms of drug-resistant bacteria such as *Candida albicans*, *P. aeruginosa*, and MRSA (Hu et al., 2022; Kaur et al., 2023). At present, the research on traditional Chinese medicine dosage forms is at a critical stage of vigorous development. Although there are numerous varieties and complex components of traditional Chinese medicine, which bring considerable challenges to the research and development work, its huge development potential is indeed worth looking forward to.

## Conclusion and prospect

The escalating problem of bacterial resistance, driven by the overuse of antibiotics, poses a significant threat to public health. This review systematically summarizes the mechanisms underlying antibiotic resistance in common bacteria across six key aspects: production of inactivating enzymes, alteration of drug targets, reduction in bacterial membrane permeability, enhanced drug efflux, induction of gene mutations, and formation of biofilms. From the QS perspective, this article elucidates the effects of active compounds, extracts, and compound formulas derived from traditional Chinese medicine against multidrug-resistant bacteria such as *P. aeruginosa*, *A. baumannii*, and *H. pylori.* This provides a strong basis for traditional Chinese medicine in reversing bacterial resistance and offers innovative strategies to address this global challenge. This article further discusses diverse delivery methods of traditional Chinese medicine and studies the differential impacts of these delivery methods on biofilms elimination. It highlights that nanotechnology may play an important role in the research and development of traditional Chinese medicine dosage forms, in the future. The research prospects of traditional Chinese medicine dosage forms are broad. However, current research perspective on QS-mediated reversal of bacterial resistance remains relatively narrow, mainly focusing on several bacteria led by *P. aeruginosa*. In the future, research on the relationship between QS and antibiotic resistance should be appropriately expanded to explore new breakthroughs in the mechanism of bacterial antibiotic resistance. Additionally, while existing literature has delineated certain mechanisms of traditional Chinese medicine in reversing resistance, the molecular mechanisms of most traditional Chinese medicine interventions remain poorly characterized. Moreover, The complex interactions among components in TCM formulas and their individual contributions to modulating bacterial resistance require further research to elucidate. In the future, research should focus on the development and utilization of traditional Chinese medicine resources and formulas, and standardize the combination of antibiotics and traditional Chinese medicine. Important attention should be paid to the following points: 1. Improve the standards for the cultivation and processing of medicinal materials, which strengthens the quality management of Chinese medicinal materials to reduce counterfeit and inferior products, and to ensure the safety and efficacy of medicinal materials. 2. Optimize the preparation process of traditional Chinese medicine prescriptions and traditional Chinese patent medicines and simple preparations, which reduces the loss of active ingredients to ensure the efficacy. Additionally, develop new traditional Chinese medicine formulations using nanotechnology, microencapsulation technology, etc., to improve the stability and targeting of traditional Chinese medicine, enhance its therapeutic efficacy, and prepare traditional Chinese medicine products with higher bioavailability and better compliance. 3. Improve toxicology research related to the combination of antibiotics and traditional Chinese medicine ingredients, which facilitates clarifying medication compatibility and avoiding serious adverse reactions. Additionally, strengthen the clinical application of combining Chinese and Western medicine to explore the best combination of antibiotics and traditional Chinese medicine, and reduce the dosage, toxic side effects, and risk of bacterial resistance of antibiotics.

We believe that with the continuous scientific advancement, the problem of bacterial resistance will be effectively addressed or alleviated.
