# Supplementary material for: Based on quorum sensing: reverse effect of traditional Chinese medicine on bacterial drug resistance mechanism
Source: Front Cell Infect Microbiol. 2025 Jun 3;15:1582003. doi: 10.3389/fcimb.2025.1582003 (PMC12188456; doi:10.3389/fcimb.2025.1582003)
Supplement: Supplementary file 1 [file DataSheet1.pdf]

Abbreviation	Full term
A. baumannii	Acinetobacter baumannii
abaI	Autoinducer synthase gene abaI
abaR	Autoinducer receptor gene abaR
AcrAB	Active efflux transporter A and B
AcrAB-TolC	Acridine resistance - AB transporter and Outer membrane channel TolC
AI-2	Autoinducers-2
AHL	Acyl-homoserine lactones
AIP	Autoinducing Peptides
methyltransferase Cfr	Chloramphenicol-florfenicol resistance methyltransferase
CydC	Cytochrome d oxidase assembly protein CydC
E. coli	Escherichia coli
EmrAB	Multidrug resistance efflux complex EmrAB
GacS/GacA	Global activator sensor/Global activator
glmU	Glucosamine-1-phosphate N-acetyltransferase and UTP-glucose-1-phosphate uridylyltransferase
gyrA	DNA gyrase (type II topoisomerase), subunit A
gyrB	DNA gyrase (type II topoisomerase), subunit B
H. pylori	Helicobacter pylori
K. pneumoniae	Klebsiella pneumoniae
Las	Lactonase-synthase system
lasI	luxI-type autoinducer synthase gene lasI
lasAB	las-regulated protease gene lasA and elastase structural genelasB
lasR	luxR-type transcriptional regulator gene lasR
lecAB	Lectin A gene and lectin B gene
LPS	Lipopolysaccharide
MacAB	Macrolide-specific efflux pump MacAB
MabTetX	Mab-type TetX
MetQ1	Methionine-binding protein MetQ1
MexXY	Multidrug efflux system MexXY-OprM
methyltransferase Cfr	Chloramphenicol-florfenicol resistance methyltransferase
M. tuberculosis	Mycobacterium tuberculosis
MRSA	Methicillin-resistant Staphylococcus aureus
murG	UDP-N-acetylmuramoyl-pentapeptide-N-acetylglucosamine transferase
MTS1438	M. tuberculosis small RNA 1438
MvfR	Multiple virulence-factor regulator
Ompk35/Ompk36	Outer membrane porins K35 and K36
Omps	Outer membrane porins
OprD	Outer membrane porins D
OqxAB	Quinolone and olaquindox efflux pump OqxAB

P. aeruginosa	Pseudomonas aeruginosa
pelABG	Pellicle-forming gene pelA, pelB, and pelG
PhzA	Phenazine biosynthesis protein A
PhzB	Phenazine biosynthesis protein B
PhzM	Phenazine biosynthesis protein M
phzABMS	Phenazine biosynthesis gene phzA, phzB, phzM, and phzS
pmrC	Polymyxin resistance gene C
PprA/PprB	Pseudomonas aeruginosa permeability regulator A/B
Pqs	Pseudomonas quinolone signal
pqsA	Pseudomonas quinolone signal gene A
pqsABCDEH	Pseudomonas quinolone signal genes pqsA, pqsB, pqsC, pqsD, pqsE, and pqsH
pqsR	Pseudomonas quinolone signal receptor gene pqsR
QS	Quorum sensing
RamA	RarA-like multidrug resistance regulator A
RamR	RarA-like multidrug resistance regulator R
Rhl	Rhamnolipid biosynthesis regulatory system
rhlI	luxI-type autoinducer synthase gene rhlI
rhlABC	Rhamnosyltransferase chain gene A, gene B, and gene C
rhlR	luxR-type transcriptional regulator gene rhlR
rpoB	RNA polymerase B subunit gene
rpsL	Ribosomal protein S12 gene
rrs	Ribosomal RNA small subunit gene
S. aureus	Staphylococcus aureus
Tet(X4)	Plasmid-mediated high-level tigecycline resistance gene
TetX	Tetracycline resistance gene
Tet(M)/Tet(S)	Tetracycline resistance gene M and S
tlyA	16S/23S rRNA (cytidine-2'-O)-methyltransferase TlyA
TolC	Outer membrane porins TolC
TPPs	Target protective proteins
TRQ	Tanreqing
UreA	Urease subunit A
UreB	Urease subunit B
